# Systemic Immunosuppression to Reduce Surgical Intervention in ANCA‐Negative Subglottic Stenosis

**DOI:** 10.1002/lary.70466

**Published:** 2026-03-04

**Authors:** Guy Benshetrit, Stephen McAdoo, Romana Kuchai, Charles Pusey, Guri Sandhu, Chadwan Al‐Yaghchi

**Affiliations:** ^1^ National Centre for Airway Reconstruction, Charing Cross Hospital, Imperial College Healthcare NHS Trust London UK; ^2^ Vasculitis Centre, Imperial College London London UK; ^3^ Vasculitis Clinic, Hammersmith Hospital, Imperial College Healthcare NHS Trust London UK; ^4^ Department of Surgery and Cancer, Faculty of Medicine Imperial College London London UK

**Keywords:** airway inflammation, ANCA‐negative vasculitis, granulomatosis with polyangiitis, idiopathic SGS, immunosuppression, subglottic stenosis

## Abstract

**Objectives:**

Idiopathic subglottic stenosis (iSGS) is a disease of unclear etiology and predictable phenotype. While surgical dilatation is the mainstay of management, a subset of patients experiences recurrent disease with minimal long‐term symptomatic relief. This study evaluates whether systemic immunosuppression alongside surgical management is an effective adjunctive treatment strategy in this patient cohort.

**Methods:**

A retrospective study was conducted on patients at a tertiary airway center with isolated SGS. The cohort was categorized into idiopathic SGS and granulomatosis with polyangiitis SGS (GPA‐SGS). A sub‐cohort of iSGS patients with aggressive disease was classified as “atypical‐SGS.” Both atypical‐SGS and GPA‐SGS cohorts received systemic immunosuppression alongside surgery and disease activity was assessed before and after immunosuppression through the inter‐dilation interval (IDI).

**Results:**

Sixty patients were included: 33 with iSGS, 20 with GPA‐SGS, and 7 with atypical SGS. The iSGS cohort had an indolent disease course, with a median IDI of 17.6 months (IQR 16.0–25.0); none received immunosuppression. GPA‐SGS patients demonstrated significantly shorter intervals prior to treatment (median 8.9 months, IQR 3.8–28.0), improving to 26.0 months (IQR 9.3–36.7) following immunosuppression (*p* = 0.0027). Similarly, in atypical SGS, IDI increased from 7.6 months (IQR 6.8–12.0) to 27.8 months (IQR 12.0–49.0) post‐treatment (*p* = 0.0496). No significant adverse events were observed.

**Conclusion:**

Atypical SGS represents a diagnostically ambiguous yet clinically aggressive subset of SGS. Systemic immunosuppression, typically reserved for GPA, may prolong disease‐free intervals in both GPA‐SGS and atypical SGS. These findings support multidisciplinary evaluation and consideration of immunotherapy in frequently recurring, ANCA‐negative SGS.

**Level of Evidence:**

3

## Graphical abstract

Idiopathic subglottic stenosis (iSGS) typically follows an indolent course managed with surgical dilatation, though a subset demonstrates aggressive, recurrent disease. In this retrospective cohort study, systemic immunosuppression significantly prolonged inter‐dilation intervals in both granulomatosis with polyangiitis‐associated SGS and an “atypical” aggressive iSGS subgroup, without significant adverse events. These findings suggest immunotherapy may be an effective adjunct in frequently recurring, ANCA‐negative SGS and support multidisciplinary evaluation in this cohort.

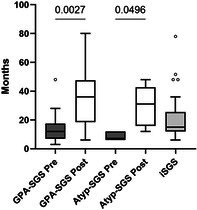

## 1 Introduction

Subglottic stenosis (SGS) refers to a disorder of excessive scar tissue deposition in the subglottic airway resulting in dyspnoea, stridor, and dysphonia. Two important aetiological subgroups include granulomatosis with polyangiitis (GPA) and idiopathic subglottic stenosis (iSGS), which are distinct disease entities with differing management strategies [1].

Categorizing diagnostic subtype is achieved through clinical, histological, and serological differentiators of disease. GPA‐driven SGS may occur as part of a syndrome with involvement of the sinonasal, pulmonary, and renal tract [2] or as a sole feature of disease confined to the upper airway. Histological features include both granulomata formation and necrotizing vasculitis, and indicative features on serology include detection of anti‐neutrophil cytoplasm antibodies (ANCA), often with specificity for proteinase 3 (PR3) [3]. The management of localized GPA‐SGS involves close collaboration between otolaryngologists and vasculitis physicians who may consider systemic immunosuppression with biologic or disease‐modifying drugs alongside surgical dilatation [4].

In contrast, iSGS is a fibroinflammatory condition of uncertain etiology, and it is generally considered to be a more quiescent disease requiring less intervention [5]. Typically, iSGS has negative autoimmune biomarker serology, and histological samples show nonspecific features of fibroinflammatory disease [6]. The most distinguishing clinical feature of the disease is the consistent and predictable phenotype [7]. It is widely established that this group of patients is almost invariably female and of Caucasian ethnicity, with estrogen considered to play a role in the disease pathogenesis [8, 9]. The management of iSGS is predominantly surgical. This is achieved through endoscopic dilatation of the stenotic segment [10, 11], often combined with local steroid injection and laser relaxing incisions. Alternative treatment options include endoscopic laser wedge resection of the scar combined with long‐term medical therapy that have been utilized in both iSGS and GPA‐SGS [12, 13, 14]. Compared to GPA‐SGS, iSGS morphology is recognized to have a more indolent course requiring fewer surgical interventions to maintain airway patency [15].

It is our experience, however, that there is a subset of patients with iSGS (with the predictable disease phenotype and negative ANCA serology typical of iSGS) but who have aggressive disease requiring multiple surgical interventions as observed in GPA. Here, we present a case series of patients with this disease morphology which we have termed “atypical SGS” (*not* as a proposed nosological category, but as a pragmatic classification to guide treatment). This cohort received systemic immunosuppression to modulate disease activity, and the effect on disease progression was assessed by measuring the disease‐free interval between surgical dilatations. This study is among the first to evaluate the efficacy of systemic immunosuppression in ANCA‐negative subglottic stenosis, a condition that remains poorly understood and challenging to manage.

## 2 Materials and Methods

### 2.1 Study Design and Approval

This is a retrospective review of patients attending a single tertiary referral unit (Imperial College NHS Foundation Trust) via the vasculitis and otolaryngology services between 2016 and 2023. The study was approved by the NHS Health Research Authority (Research Ethics Committee [REC] reference 20/WS/0181). Adult patients with a diagnosis of SGS were identified by searching electronic patient databases. Patients who did not have a diagnosis of either idiopathic or ANCA‐associated subglottic stenosis were excluded from the study, including pediatric and other congenital or acquired causes of disease.

Demographic data of age, sex, ethnicity, age at onset of symptoms and comorbidities were collected by electronic chart review. Location and extension of stenosis, serology and biopsy results, number and interval of endoscopic dilations before and after the commencement of systemic treatment were also collected. In addition, details of systemic treatments were documented for the GPA‐SGS and Atypical‐SGS cohorts.

### 2.2 Disease Cohorts

**FIGURE 1:**
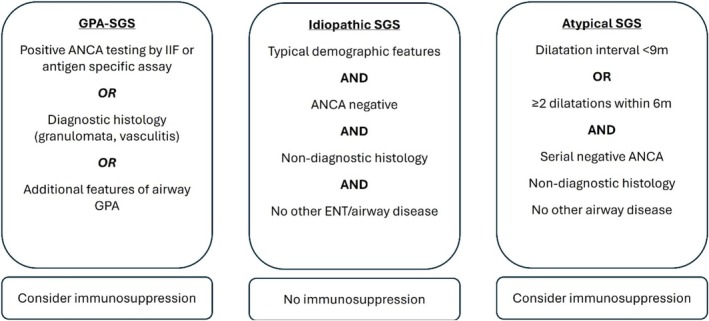
Detailed inclusion criteria by disease subgroup. ANCA, antineutrophil cytoplasmic antibodies; GPA, granulomatosis with polyangiitis; IIF, indirect immunoflourescence; SGS, subglottic stenosis.

The included patients were categorized into three groups to allow comparison of clinical and treatment‐related variables. A visual summary of the classification criteria used to define these three subgroups is shown in Figure 1.

- *Idiopathic SGS* was diagnosed following assessment by an experienced clinician. Criteria for diagnosis included typical patient demographic features and disease course, serially negative ANCA, subglottic biopsy with no histological evidence of vasculitis, and no intubation in the preceding 2 years.
- *GPA‐SGS* were those presumed to have a diagnosis of localized GPA affecting the airway. All patients had SGS as the sole or predominant feature of their disease with no other systemic features of vasculitis such as pulmonary, renal or ocular involvement. Inclusion criteria for this cohort included any one of (i) any positive ANCA serology on serial measurement; (ii) granulomata or overt vasculitis on histology; (iii) evidence airway disease beyond the subglottis (e.g., tracheal disease). Serological testing for ANCA was performed by indirect immunofluorescence assay (IIF) and enzyme‐linked immunosorbent assay (ELISA). This group was referred to the vasculitis service for consideration of immunosuppression.
- *Atypical‐SGS* had features of both idiopathic and GPA‐associated disease but did not meet the essential criteria of either condition. Inclusion criteria for this cohort included all of the following: (i) airway disease limited to the subglottis; (ii) nondiagnostic histology; (iii) serially negative ANCA serology on at least three occasions; and (iv) inter‐dilatation intervals (IDI) of < 9 months or ≥ 2 surgical interventions within 6 months. Due to frequent airway intervention, these patients were offered immunosuppression following review in a combined ENT‐vasculitis service.

### 2.3 Surgical Interventions

Our standard surgical approach involves suspension microlaryngoscopy and supraglottic high‐frequency jet ventilation, Methylprednisolone acetate (40 mg/mL) injection and three releasing incisions at 12, 4, and 7 O'clock using carbon dioxide laser. The stenotic segment is then dilated with a semi‐compliant airway‐dilation balloon. This is then followed by two biopsies, superficial and deep, using standard endoscopic cup forceps and curved scissors of the Bouchayer Laryngeal Instruments set (Integra MicroFrance, York, PA, USA).

### 2.4 Statistical Analysis

Data are reported using parametric (mean, SD) and nonparametric (median, IQR) summary statistics as appropriate. Comparison between groups was by Fisher exact test for categorical data, and by 1‐way ANOVA and Kruskall–Wallis tests for parametric and nonparametric continuous variables, respectively. Paired *t*‐tests were used to compare pre‐ and post‐immunosuppression interdilation intervals in both the GPA‐SGS and atypical‐SGS cohorts, using GraphPad Prism 10.3.0.

## 3 Results

We identified 60 patients who met the inclusion criteria for this study (Table 1). All patients were initially referred to the tertiary airway service at ICHNT with subglottic stenosis as the sole or predominant feature of disease. The two largest groups were idiopathic‐SGS patients, comprising 33 cases (55%), and the GPA‐SGS group (*n* = 20, 33%). A total of 7 cases (12%) met criteria for atypical‐SGS.

**TABLE 1:** Clinical features of patients presenting with subglottic stenosis.

	Idiopathic‐SGS	GPA‐SGS	Atypical‐SGS	*p*‐Value
Number	33	20	7	
Sex, *n* (%) female	33 (100%)	15 (75%)	6 (86%)	0.012
Stenosis location *n* (%)				< 0.01
SGS only	33 (100%)	11 (55%)	5 (71%)	
Glottic extension	0 (0%)	1 (5%)	1 (14.5%)	
Tracheal	0 (0%)	8 (40%)	1 (14.5%)	
Non‐Caucasian ethnicity	0	4 (20%)	1 (14%)	0.032
Age at onset, years (IQR)	49 (27.2–53.4)	45 (28.0–61.2)	42 (37.3–53.3)	< 0.01
Duration of follow up, months	82 (36–120)	77 (60–96)	73 (57–90)	< 0.01

Abbreviations: GPA, granulomatosis with polyangiitis; SGS, subglottic stenosis.

The mean age of onset was in the 5th decade in all groups; however, patients with atypical‐ and GPA‐SGS tended to present earlier than patients with idiopathic‐SGS. As expected, all patients in the idiopathic‐SGS cohort were female and of caucasian ethnicity; female preponderance was also observed in the GPA‐ and atypical‐SGS groups but not exclusively. The average duration of follow up was comparable in all groups (73–82 months).

### 3.1 Idiopathic SGS

All patients in this group had isolated subglottic stenosis with no other clinical findings indicating a diagosis of GPA. None had positive ANCA serology on repeated laboratory testing. All patients underwent at least one subglottic biopsy; histopathological findings included acute and chronic inflammation and dense fibrosis, but none had pathognomic features of necrotizing vasculitis or granulomata. The median interdilation in this group was 17.6 months (IQR 16.0–25.0).

### 3.2 GPA‐SGS

All patients had subglottic stenosis as the sole or predominant disease feature, and none had evidence of systemic vasculitis beyond the respiratory tract. In addition to SGS, eight patients demonstrated more distal tracheal stenosis, and one patient had extension proximally to the glottis (45%). The most common findings on biopsy were nondiagnostic features of active and chronic inflammation (*n* = 14, 70%); while six patients (30%) had histopathological features of necrotizing vasculitis or granuloma formation in keeping with GPA‐SGS. A total of 19/20 (95%) patients had positive ANCA serology, thus meeting criteria for GPA‐SGS (with or without other diagnostic clinical or histopathological features of GPA‐SGS). A summary of ANCA serology by both indirect immunofluorescence (IIF) and antigen‐specific assays (ELISA) is provided in Table 2. The median interdilation interval, prior to receiving immunosuppression, was 8.9 months (IQR 3.8–28.0).

**TABLE 2:** ANCA serology by IIF and ELISA in the GPA‐SGS cohort.

IIF assay	pANCA	cANCA	Negative
No of patients	4	16	1

ELISA assay	MPO	PR3	Negative
No of Patients	2	5	14

Abbreviations: ELISA, enzyme linked immunosorbent assay; IIF assay, indirect immunoflourescence assay; MPO, myeloperoxidase; p/cANCA, peripheral/central antineutrophil cytoplasmic antibodies; PR3, Proteinase 3.

### 3.3 Atypical SGS

**FIGURE 2:**
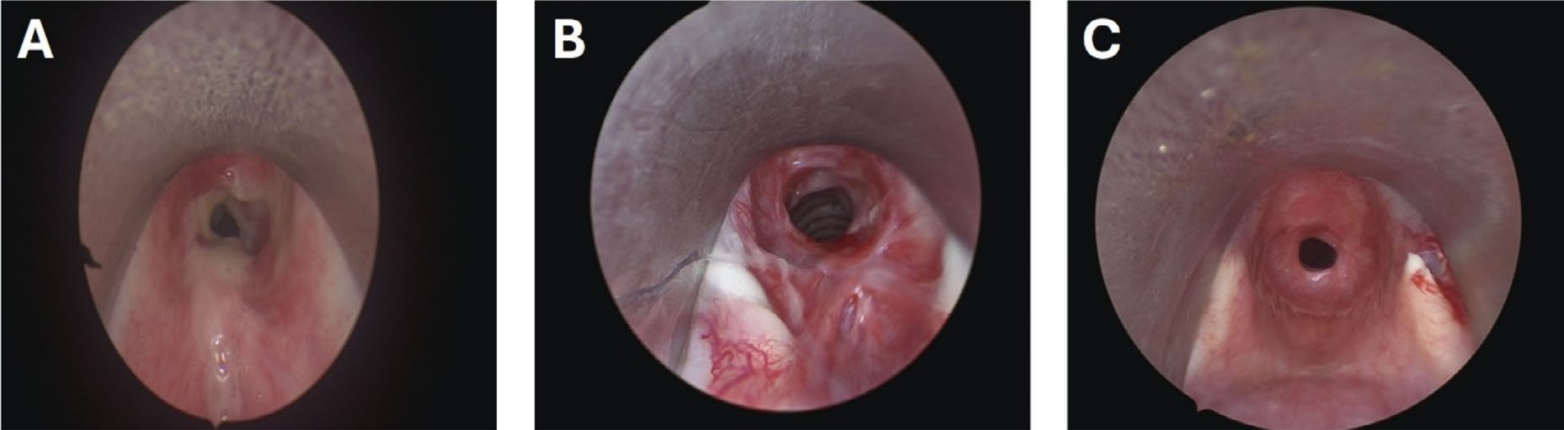
Endoscopic views of the larynx in each subgroup (A) GPA‐SGS with evidence of acute inflammation; (B) active disease in atypical SGS; (C) 50% circumferential scarring and stenosis in idiopathic SGS.

We identified seven cases who met our classification criteria for idiopathic SGS, including negative ANCA serology and nondiagnostic histology, but who required frequent airway intervention to maintain patency. Demographic details, disease durations, number and nature of interventions and comorbidities are presented in Table 3. The average IDI, prior to receiving immunosuppression, was 7.6 months (6.8–12.0). These patients tended to have inflammatory airway appearances at endoscopy (Figure 2); otherwise, there was no difference in disease location, with one patient having proximal extension of stenosis. All seven patients agreed to the proposed immune suppression as a treatment escalation option rather than opting for open or endoscopic reconstruction.

**TABLE 3:** Clinical features of atypical SGS cohort.

	Age	Age at onset	Disease duration (years)	No of pretreatment interventions	Intervention	Comorbidities
Patient 1	69	54	15	8	Steroid, laser, balloon	Ischemic heart disease
Patient 2	64	54	10	4	Steroid, laser, balloon	Nil
Patient 3	65	53	12	6	Steroid, laser, balloon	Obesity, hypertension
Patient 4	55	42	13	3	Steroid, laser, balloon	Nil
Patient 5	62	38	24	6	Steroid, laser, balloon	Obesity
Patient 6	57	38	19	3	Steroid, laser, balloon	Nil
Patient 7	43	37	6	4	Steroid, laser, balloon	Nil
Mean	62	42	13	4		
IQR	(56–64.5)	(38–53.5)	(11–17)	(3.5–6)		

### 3.4 Interdilatation Interval and the Effect of Immunosuppression

**FIGURE 3:**
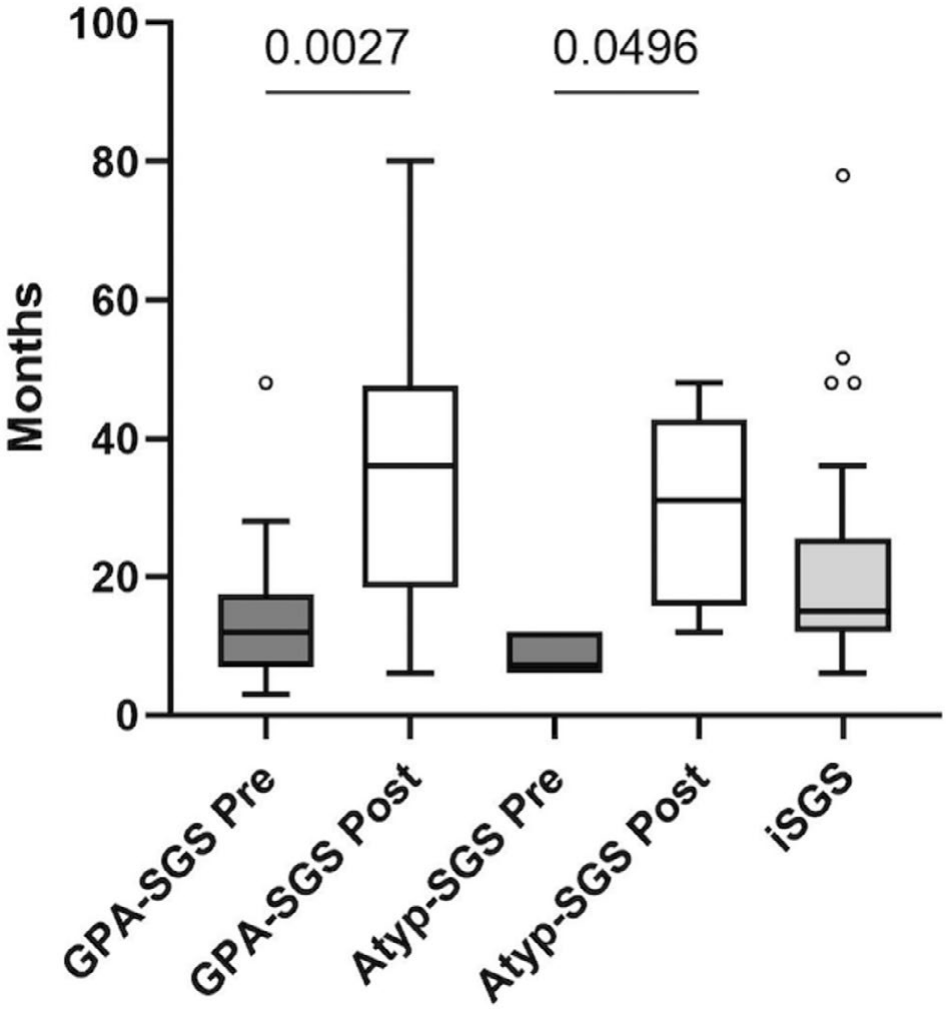
Pre‐ and post‐treatment inter‐dilation interval in GPA‐ and atypicalSGS. Boxplot illustrating the inter‐dilation interval in months (*y* axis) for the granulomatosis with polyangiitis—subglottic stenosis group pre (GPA‐SGS Pre) and post (GPA‐SGS Post) Immunosuppression. The same comparison was made for the atypical subglottic stenosis group (Atyp‐SGS Pre, Atyp‐SGS Post) and the idiopathic subglottic stenosis (iSGS) group.

Patients with a definitive diagnosis of idiopathic‐SGS had the longest IDI, at 17.6 months (IQR 16.0–25.0), and none of these cases were treated with immunosuppression. Given the frequency of airway intervention, immunosuppression was administered to both the GPA‐SGS and atypical‐SGS cohorts (Table 4). In the GPA‐SGS cohort, the IDI increased from 8.9 (3.8–28.0) to 26.0 (9.3–16.7) months (*p* = 0.0027). A comparable increase was observed in the atypical‐SGS group with average IDI rising from 7.6 (6.8–12.0) to 27.8 (12.0–49.0) months (*p* = 0.0496). Thus, both the GPA‐SGS and atypical‐SGS cohorts demonstrated significant increases in IDI following systemic immunosuppression, suggesting its efficacy in reducing disease recurrence (Figure 3). There were no severe infections or mortality identified in relation to the immunosuppression.

**TABLE 4:** Immunosuppression use in patients with atypical and GPA‐SGS.

	Atypical‐SGS (*n* = 7)	GPA‐SGS (*n* = 20)	*p*‐Value
Induction treatment			
Prednisolone alone	1 (14%)	3 (13%)	1
Prednisolone as adjunct	5 (71%)	12 (50%)	0.757
Azathioprine	1 (14%)	10 (41%)	0.262
Rituximab	2 (28%)	1 (4%)	0.17
Methotrexate	2 (28%)	Nil	0.067
Mycophenolate Mofetil	1 (14%)	1 (4%)	0.464
Cyclophosphamide	Nil	5 (21%)	NA

## 4 Discussion

Idiopathic SGS and GPA are separate disease entities with clinical features unique to each disease cohort. Translational studies reveal distinct mechanisms for iSGS and granulomatosis with polyangiitis (GPA). iSGS appears driven by epithelial dysfunction leading to microbiome disruption, immune dysregulation, and localized fibrosis [16, 17]. In contrast, GPA is strongly linked to HLA Class II, particularly the HLA–DPB1*04 allele, with risk allele expression localized to immune cells [18, 19]. Notably, iSGS shows no HLA association [20], supporting its distinct, epithelial‐centered pathogenesis.

The differentiation of idiopathic SGS from airway‐localized GPA can, however, be difficult in clinical practice, and this study is among the first to address challenges in managing subglottic stenosis patients who develop frequent recurrence requiring repeated airway intervention without clear evidence of GPA.

In our study, all patients with idiopathic disease were female and Caucasian, with a mean age of onset of 49 years old. This is consistent with existing data which consistently depict idiopathic SGS as a disease with a highly predictable phenotype and perimenopausal onset [7, 11]. Likewise, serial ANCA testing was consistently negative and histological analysis revealed only features of chronic inflammation and fibrosis.

Twenty patients were included in the GPA‐SGS cohort, where typical disease features were again noted. Female predominance was observed (73.9%), which is typical of airway‐limited GPA [21] compared to patients with multisystem disease. The most common ANCA serotype was cANCA/PR3‐ANCA (80%), which is consistent with large cohort studies of isolated upper airway or head and neck‐limited disease [22, 23]. We note that a significant proportion (70%) of cases were positive by IIF assay only, with negative antigen‐specific testing by ELISA, suggesting IIF testing remains an important diagnostic tool in the setting of localized airway disease [24]. Systemic immune suppression in our cohort led to a significant improvement in the IDI from 8.9 to 26.0 months on average. The role of systemic therapy in managing GPA‐SGS remains debated, as airway disease can progress independently of systemic inflammation and may be refractory to conventional immunosuppressive or biologic treatment [1], with Lally et al. reporting no difference in subglottic inflammation between rituximab‐treated and untreated patients [25]. Consistent with our results, Moroni et al. reported that systemic immunosuppression, in particular rituximab and cyclophosphamide, provides benefit in preventing recurrence of SGS in GPA patients [22]. The only standardized medication across all patients was corticosteroid. The heterogeneity of other adjunctive immunosuppression reflects evolving clinical practices and a lack of standardized treatment protocols for SGS. While this study is underpowered to compare the efficacy of individual agents, we are currently investigating these effects in a much larger patient cohort to better define optimal systemic treatment strategies.

We identified seven patients who exhibited overlap features of both idiopathic and GPA‐associated disease, and therefore categorization into either subtype presented a clinical challenge. Each of these patients had aggressive disease with an IDI of between 6 and 12 months, which is more in keeping with GPA‐SGS; however, their negative serology, histology, and lack of other organ involvement introduced diagnostic uncertainty.

Seronegative ANCA‐associated vasculitis is a well‐recognized phenomenon [26]. The prevalence of this disease pattern is particularly high in localized GPA [27] and has been reported in 11%–31% of cases of ENT‐limited disease [28, 29]. Likewise, it is well recognized that biopsy can yield a high false‐negative rate particularly in the upper respiratory tract [30] and it is therefore an equally unreliable differentiator of disease. It is possible, therefore, that our patients categorized as atypical‐SGS represent a group with ANCA‐negative GPA and highlight that—given their favorable response to immunosuppressive treatment—a lack of positive serology or confirmatory histology should not preclude referral to vasculitis physicians and consideration for systemic immunotherapy.

Alternatively, our atypical‐SGS cohort may represent an aggressive variant of idiopathic SGS. Large case series have now reported similar non‐GPA cohorts undergoing more than 10 endoscopic dilatations [13, 14] in the most difficult to manage cases. Several adjuvant treatment therapies have been trialed in non‐GPA‐SGS including proton pump inhibitors and antibiotic therapies [31], which have had modest success in modulating disease activity. The role of immunosuppression in non‐GPA‐SGS has, to date, predominantly focused on localized injection of corticosteroids at the time of surgery [32, 33, 34] or a series of transcutaneous intralesional steroid injections. This study is among the first to report on a cohort of frequently‐recurring SGS patients receiving systemic disease‐modifying therapies as a key feature of their management strategy. All seven patients received a course of high‐dose oral steroids alongside variable disease‐modifying or biologic therapy in the form of rituximab. Despite the small sample size of 7 patients, a statistically significant increase in the average IDI from 7.6 to 27.8 months suggests that the addition of systemic immunosuppression was an effective treatment modality in this challenging population. Five of the 7 patients required at least one more dilation procedure during the follow up period, and the two that did not were followed up for shorter periods of 11 and 18 months, respectively, after initiation of immunosuppression. There were no significant drug side effects documented within the follow up period.

Our study investigating the use of immunosuppression in atypical‐SGS complements other work investigating systemic treatment in idiopathic SGS. To date, there are only two documented case series testing immunotherapy in a similar cohort with seronegative disease with < 12 months between dilation procedures [34, 35]. Induction regimens of either single or combination methotrexate and rituximab were observed to increase the time between dilations from a median of 338 days to 697 [35]. Similarly, in a Phase 1 trial using the mTor inhibitor everolimus, induction therapy on the day of surgery had a sustained increase in peak flow measurements six months post operatively with minimal adverse side effects [36]. Our study adds valuable evidence to the limited data on systemic immunosuppression in atypical SGS, demonstrating its potential to significantly prolong dilation intervals and improve symptom relief without significant adverse effects.

Access to appropriate diagnosis and immune suppression therapy remains an unmet need for this patient cohort. Within our center, suspected and confirmed vasculitis patients with ENT manifestations, including atypical‐SGS, are managed through a long‐standing Joint ENT/Vasculitis service. Monthly combined clinics allow for shared review of patients, facilitate early identification of atypical or vasculitis‐associated features, and support timely initiation of treatments. We recognize that there is a general reluctance among vasculitis physicians to offer immune suppression for seronegative or atypical SGS patients. While joint clinics may not be a feasible option in low‐volume centers, bi‐speciality case conferences might overcome some of these barriers and allow shared understanding of treatment challenges on both sides. To further guide clinicians, including those early in their careers, the senior authors (SMC and CAY) have included a recommendation to refer atypical SGS cases to a vasculitis service in the 2025 British Society for Rheumatology Management Recommendations for ANCA‐associated Vasculitis [37].

## 5 Limitations

Limitations of our work include the small sample size of seven patients in the atypical‐SGS group. This likely reflects the rarity of this disease subtype, given the high case volume of patients with subglottic stenosis referred to our center. The retrospective nature of this study also contributed to the lack of standardized treatment protocols. Furthermore, systematic, consistent documentation of peak flow data was lacking in the charts, precluding any meaningful comparison as an additional objective outcome measure.

Larger, prospective multi‐center studies are required to fully characterize the clinical phenotype of patients with SGS and to assess defined treatment protocols against one another. These may also provide the basis to investigate underlying disease mechanisms and to develop improved classification and diagnostic criteria for variant presentations of subglottic stenosis.

## 6 Conclusion

This study is among the first to address the difficulties in managing patients with subglottic stenosis who develop frequent disease recurrence without clear evidence of GPA. Although they represent a small proportion of the total subglottic stenosis population, patients with atypical‐SGS experience significant symptom burden, and the current medical and surgical treatment modalities are ineffective in preventing disease recurrence. Our experience suggests that careful multidisciplinary collaboration between ENT and vasculitis specialists is essential to differentiate patients with localized forms of GPA and to identify those with atypical forms of SGS who may benefit from immunosuppressive therapy.

While systemic immunotherapy could theoretically prolong interdilation intervals even in individuals with “typical” iSGS, current evidence does not support its routine use outside a formal clinical trial. The balance between potential benefit and treatment‐related risk remains uncertain, and therefore, widespread application cannot be recommended at this stage. Our ongoing research program is focused on identifying serological and histological markers that can more reliably distinguish atypical SGS and identify those patients most likely to benefit from immune suppression. These findings will inform the design of future clinical trials aimed at evaluating whether the use of immunotherapy should be expanded within the wider iSGS population.

## Funding

The authors have nothing to report.

## Conflicts of Interest

The authors declare no conflicts of interest.

## Data Availability

The data that support the findings of this study are available from the corresponding author upon reasonable request.
